# The Relationship Between C-Reactive Protein–Triacylglycerol–Glucose Index and All-Cause Mortality in Patients With Cardiovascular Disease: A Retrospective Cohort Study and Development of a Machine Learning Prediction Model

**DOI:** 10.1155/cdr/6914985

**Published:** 2025-09-03

**Authors:** Wenlong Ding, Fachao Shi, Zheng Wang, Long Wang, Caoyang Fang

**Affiliations:** ^1^Department of Cardiology, Xuancheng Hospital Affiliated to Wannan Medical College (Xuancheng People's Hospital), Xuancheng, Anhui, China; ^2^Department of Cardiology, Maanshan People's Hospital, Maanshan, Anhui, China; ^3^Department of Cardiology, The Second People's Hospital of Hefei, Hefei Hospital Affiliated to Anhui Medical University, Hefei, Anhui, China; ^4^Department of Emergency, The First Affiliated Hospital of USTC, Division of Life Sciences and Medicine, University of Science and Technology of China, Hefei, Anhui, China

**Keywords:** all-cause mortality, cardiovascular disease, C-reactive protein-triglyceride-glucose index, CTI, CVD, machine learning

## Abstract

**Objective:** The CTI is increasingly recognized as a new marker for assessing inflammation and insulin resistance. However, the relationship between CTI and all-cause mortality risk in patients with CVD remains unclear.

**Methods:** We analyzed data from the NHANES from 1999 to 2010. The correlation between CTI and all-cause mortality risk in CVD patients was examined using Cox regression analysis. Nonlinear relationships between CTI and all-cause mortality risk were explored through restricted cubic splines and Cox proportional hazards regression. We employed six ML models, including RF, LightGBM, DT, XGBoost, LR, and KNN, to predict all-cause mortality risk in CVD patients based on CTI and SHAP for interpretability.

**Results:** A total of 1429 CVD patients were included, with 849 all-cause deaths recorded during the follow-up period. After adjusting for potential confounding factors, the highest quartile of CTI (Q4) significantly increased the risk of all-cause mortality compared to the lowest quartile (Q1) (HR = 1.38, 95% CI: 1.04–1.84, *p* = 0.03). Restricted cubic splines demonstrated a nonlinear relationship between CTI and all-cause mortality risk in CVD patients. Among the machine learning models, the LightGBM model exhibited the best predictive performance, with an ROC of 0.967, accuracy of 0.909, sensitivity of 0.906, specificity of 0.914, *F*1 score of 0.922, recall of 0.906, and PR of 0.979. SHAP analysis identified age, BU, and CTI as the primary predictive factors, with CTI positively correlated with all-cause mortality risk in CVD patients.

**Conclusion:** There is a nonlinear relationship between CTI and all-cause mortality risk in CVD patients, with elevated CTI levels significantly associated with increased mortality risk. Additionally, for the first time, this study constructed a machine learning model to predict all-cause mortality risk in cardiovascular disease using CTI, with LightGBM demonstrating the best predictive performance. SHAP analysis identified age, BUN, and CTI as critical factors in the prediction, providing valuable references for future related research.

## 1. Introduction

Cardiovascular disease (CVD) is one of the leading causes of death worldwide, with its incidence and mortality rates continuing to rise, posing significant challenges to public health. According to statistics from the World Health Organization, CVD is responsible for approximately 17 million deaths each year, accounting for 31% of all global deaths. Effective risk assessment and prognosis prediction are crucial for improving the management of CVD patients. Traditional assessments of CVD risk primarily rely on conventional risk factors such as age, gender, blood pressure, and lipid levels [1–3]. However, these factors have certain limitations in explaining individual variability and predicting outcomes [4]. Therefore, it is essential to identify new biomarkers to enhance the accuracy of risk assessments.

In recent years, inflammation and metabolic abnormalities have been recognized as significant pathological mechanisms underlying CVD. CRP, a sensitive inflammatory marker, has been extensively studied and is closely associated with the occurrence of cardiovascular events. Research has shown that elevated CRP levels correlate with an increased risk of atherosclerosis, myocardial infarction, and other cardiovascular events [5–7]. Furthermore, the TyG has emerged as a promising indicator of insulin resistance, demonstrating potential value in assessing metabolic syndrome and CVD risk. The calculation of the TyG index is straightforward, and it exhibits a strong correlation with insulin resistance, proving to be effective in predicting cardiovascular events [8, 9].

Although existing risk prediction tools (such as the Framingham risk score and SCORE system) [10] have demonstrated value in predicting cardiovascular events, they have limitations in CVD, particularly when considering cardiovascular inflammation index (CTI) as an emerging biomarker. Our study is aimed at addressing the following clinical gaps through machine learning approaches: (1) developing a high-precision risk stratification tool that integrates traditional risk factors and CTI to more accurately identify high-risk patients, (2) providing clinicians with a decision support system to guide early intervention strategies and follow-up intensity, and (3) optimizing resource allocation by directing more intensive monitoring and aggressive treatment regimens to patients most likely to benefit. The advantage of machine learning models lies in their ability to capture complex nonlinear relationships and interactions between variables, which are often difficult to achieve with traditional statistical methods. This study is aimed at exploring the relationship between CTI and overall mortality in CVD patients through a retrospective cohort analysis and establishing a predictive model based on machine learning methods. This research holds significant clinical implications.

## 2. Methods

### 2.1. Data Source and Study Population

The NHANES is an ongoing research initiative aimed at assessing the health status of the US population. The study has received approval from the National Center for Health Statistics Ethics Review Board, and all participants provided written informed consent. Therefore, the Ethics Committee of the Fourth Affiliated Hospital of Soochow University exempted this research from ethical review. The data for this study were sourced from the NHANES database covering the years 1999–2018.

**Figure 1:**
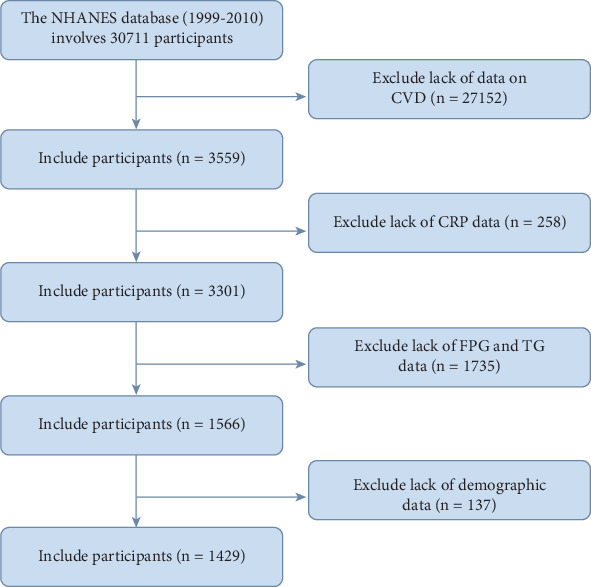
Study flow chart.

This study analyzed data from 30,711 participants aged 18 and older between 1999 and 2010. Exclusion criteria included participants without CVD data (27,152 individuals); those lacking data on CRP, fasting blood glucose, and triglycerides (TGs) (1993); and individuals lacking demographic information such as marital status, education, alcohol consumption habits, and diabetes (137). The missing rate for other continuous variables was below 10%, and we employed the “MICE” method for multiple imputation. Ultimately, a total of 1429 eligible participants were included for analysis. The study workflow is illustrated in Figure 1.

### 2.2. Diagnosis of CVD

The diagnosis of CVD was conducted through standardized medical questionnaires during personal interviews, where participants confirmed their disease status via self-reported diagnoses. Interviewers asked participants whether they had ever been diagnosed with conditions such as coronary artery disease, congestive heart failure, angina, myocardial infarction, or stroke. If a participant answered “yes,” they were considered to have CVD.

### 2.3. Measurement of CTI

The formula for calculating the CTI [11] is 0.412∗Ln(CRP) + TyG, where TyG is defined as Ln[fasting TGs (mg/dL) × fasting glucose (FPG) (mg/dL)/2] [12]. Blood samples were collected in the morning after an 8.5-h fasting period and sent to laboratories certified by the National Center for Health Statistics for processing. Serum TG levels were measured using the Roche Modular P and Roche Cobas 6000 chemical analyzers, while FPG levels were determined using the oxygen rate method with the Beckman dx800 analyzer. Additionally, CRP levels were quantified using a latex-enhanced turbidimetric assay method with a Behring turbidimeter. For more detailed information on laboratory testing, please refer to the NHANES official website. In general, higher CTI values indicate a more severe degree of inflammation and insulin resistance [13].

### 2.4. Covariates

This study considered a variety of covariates, including age, sex, race, education, marital status, and poverty income ratio (PIR), as well as information on hypertension, BMI, smoking, and alcohol consumption. Racial categories included non-Hispanic Black, non-Hispanic White, Mexican American, and others. The PIR was divided into three categories: less than 1.3, between 1.3 and 3.5, and greater than 3.5. Marital status was classified as married, divorced, unmarried, and other. Smoking status was categorized into three groups: (1) never smoked, (2) formerly smoked, and (3) currently smoking. Alcohol consumption was classified into five categories: never drank, formerly drank but no longer, heavy drinking, moderate drinking, and light drinking. The laboratory test indices included creatinine, uric acid, blood urea nitrogen, fasting glucose, HbA1c, CRP, TG, TC, HDL, and LDL, among others.

### 2.5. Subgroup Analysis

To comprehensively assess the impact of various variables, this study conducted subgroup analyses based on several classification criteria, including age (under 60 years and 60 years or older), gender (male and female), race (Mexican American, non-Hispanic White, non-Hispanic Black, and others), PIR (< 1.3, 1.3–3.5, and > 3.5), and hypertension and diabetes status.

### 2.6. Statistical Analysis

#### 2.6.1. Basic Analysis and Regression Analysis

This study conducted data analysis using R software (Version 4.3.2, https://www.r-project.org). We adhered to the NHANES analysis and reporting guidelines and took into account the complex sampling design and weights. In the weighted analysis, we utilized the MEC sample weights. Participants were categorized into four groups based on the quartiles of the CTI (Q1–Q4). Continuous variables are presented as means (SE), while categorical variables are displayed as frequencies (percentage). To compare the baseline characteristics of participants across different CTI quartiles, we performed one-way ANOVA and Pearson's chi-square tests to examine differences in continuous and categorical variables. For investigating the relationship between CTI and all-cause mortality risk among CVD patients, we employed multivariable Cox proportional hazards regression models, establishing three models: Model 1 was unadjusted; Model 2 adjusted for age, race, and gender; and Model 3 adjusted for age, race, gender, marital status, educational level, hypertension, diabetes, PIR, uric acid, BMI, HbA1c, TC, HDL, and LDL. Covariate selection was based on the following principles: (1) established risk factors for cardiovascular outcomes identified in published literature, (2) variables showing clinical relevance to both CTI and mortality, and (3) avoidance of overadjustment and multicollinearity issues in the model. When assessing the dose–response relationship between CTI and mortality, we applied restricted cubic splines and smoothed curve fitting in the Cox proportional hazards regression model to explore nonlinear associations. To visually assess differences in survival across CTI quartiles, the Kaplan–Meier survival curves were plotted, and log-rank tests were performed to evaluate statistical differences in survival distributions between groups. In addition, LASSO regression was used to screen for characteristics associated with the risk of all-cause mortality in patients with CVD and included in subsequent machine learning analyses.

#### 2.6.2. Construction of Machine Learning Models

In this study, we stratified the data based on all-cause mortality outcomes for CVD patients and randomly divided the dataset into a training set and a testing set in a 7:3 ratio. The training set was used to calculate and train the model parameters, while the testing set was employed to evaluate model performance. We selected six machine learning models, including RF, LightGBM, DT, XGBoost, LR, and KNN, to predict the all-cause mortality risk in CVD patients. To address the class imbalance between the mortality and nonmortality groups, we applied synthetic minority oversampling technique to generate synthetic samples for the minority class, thereby optimizing model performance [14]. Additionally, to prevent overfitting and enhance the generalizability of the models, we employed 10-fold cross-validation for evaluation and constructed the final model after repeated iterations. Through grid search, we explored all parameter combinations to identify those that provided the best overall performance. Model performance was assessed using metrics such as ROC, accuracy, sensitivity, specificity, *F*1 score, recall, and precision–recall.

#### 2.6.3. Interpretation of Machine Learning Models

By applying the SHAP (Shapley's Additive Explanations) algorithm, we calculated the SHAP values for each variable, ultimately identifying the best performing model. SHAP is a machine learning prediction methodology rooted in game theory, providing a unified framework for interpreting machine learning predictions and serving as a novel approach for analyzing various complex “black box” models [15]. Each feature within a sample corresponds to a SHAP value, where a positive value indicates that the feature contributes positively to the predicted probability, while a negative value indicates a negative impact. Consequently, the weighted average of all SHAP values reflects the overall influence of features on the predictive model. We also visualized the relationship between features and the predictive model through global feature importance plots and beeswarm plots.

## 3. Results

### 3.1. Baseline Characteristics of Participants

This study included a total of 1429 participants, with a mean age of 64.45 (0.46) years, of which 55.67% were male. The results indicate that the average age of the mortality group was significantly higher than that of the survival group (*p* < 0.0001), suggesting that age is an important prognostic factor. Furthermore, levels of creatinine and BUN were significantly elevated in the mortality group (*p* < 0.0001), and the CTI levels were also significantly higher compared to the survival group (*p* = 0.01). Statistical significance (*p* < 0.05) was achieved in comparisons between the two groups regarding HDL, BMI, HbA1c, race, marital status, drinking habits, and the presence of hypertension and diabetes; however, no significant differences were observed in other indicators. Detailed data can be found in Table 1.

### 3.2. The Relationship Between CTI and All-Cause Mortality

**Figure 2:**
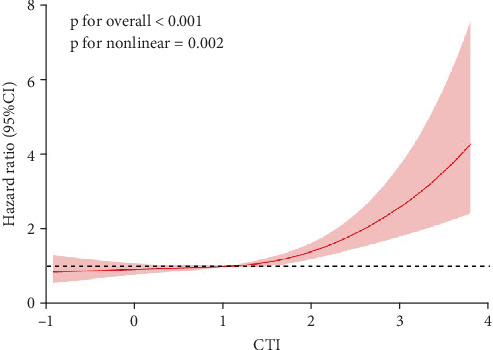
RCS analysis of association of CTI with all-cause mortality.

**Figure 3:**
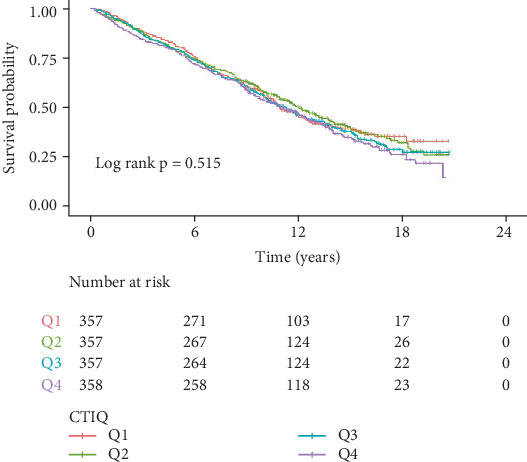
Kaplan–Meier survival curves for patients with CVD in different CTI groups. *p* < 0.0001 by log-rank test.

During an average follow-up period of 10.08 years, a total of 849 cases of all-cause mortality were observed.  Table 2 presents the relationship between CTI and both all-cause mortality and cardiovascular mortality. We employed a weighted multivariable Cox proportional hazards regression model to analyze the independent association between CTI and mortality risk. In the fully adjusted Model 3, a significant positive correlation was found between CTI and all-cause mortality risk in CVD patients (HR = 1.24, 95% CI: 1.07–1.44, *p* = 0.004). Using the lowest quartile (Q1) as a reference, the highest quartile (Q4) significantly increased the all-cause mortality risk for CVD patients (HR = 1.38, 95% CI: 1.04–1.84, *p* = 0.03). Additionally, we conducted a smooth curve fitting to explore the relationship between CTI and both all-cause mortality and cardiovascular mortality rates. The results, illustrated in Figure 2, indicate a nonlinear relationship between CTI and risks of all-cause and cardiovascular mortality.  Figure 3 presents the Kaplan–Meier survival curves stratified by CTI quartiles. Although visual inspection suggests a trend toward lower survival probability with increasing CTI quartiles, the log-rank test indicated that these differences did not reach statistical significance (*p* = 0.515). This suggests that while there may be a relationship between CTI and mortality as demonstrated in our Cox regression models after adjusting for confounders, the unadjusted survival differences alone are not statistically significant.

### 3.3. Subgroup Analysis

**Figure 4:**
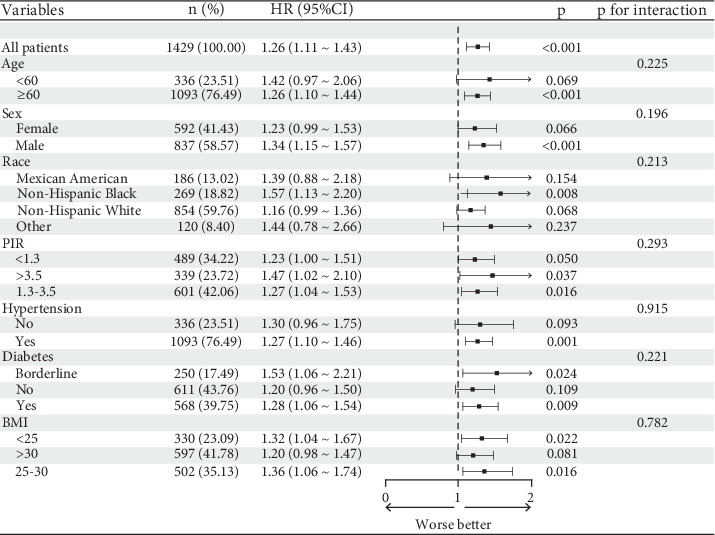
Forest plot for subgroup analyses.

In the subgroup analysis presented in Figure 4, we stratified the data according to factors such as age, gender, race, PIR, hypertension, and diabetes. The results demonstrated that the relationship between CTI and all-cause mortality risk remained consistent across all subgroups. Furthermore, no significant interactions were observed among the stratified variables (*p* > 0.05).

### 3.4. Important Factors for Screening the Risk of All-Cause Mortality in Patients With CVD by LASSO Curve

**Figure 5:**
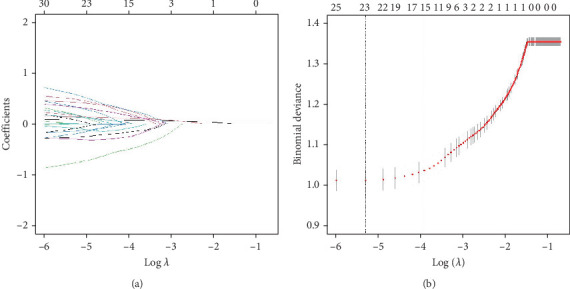
Presentation of the results of the LASSO regression analysis. (a) LASSO regression model screening variable trajectories. (b) LASSO regression model factor selection: left dashed line represents the optimal lambda value (lambda.min), while the right dashed line marks the lambda value within one standard error of the optimal (lambda.1se).

To select the most suitable variables, LASSO regression was applied. As shown in Figure 5a, with an increase in log (*λ*), the coefficients in the model gradually approach zero, indicating that the independent variables are eliminated one by one. In Figure 5b, it can be observed that all variables are excluded at lambda.1se, leaving no coefficients in the model; whereas at lambda.min, the LASSO regression model retains 21 variables and achieves the lowest prediction error. Based on 10-fold cross-validation, LASSO ultimately identified age, sex, race, marital status, smoke, CTI, BUN, creatinine, BMI, and UA as the optimal independent variables in the training set.

### 3.5. Comparison of Machine Learning Models

**Figure 6:**
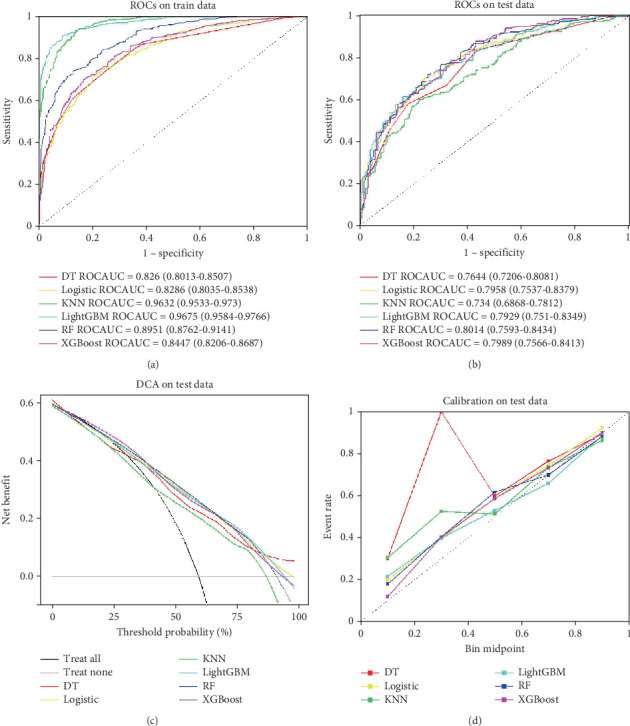
(a–d) ROC analysis, calibration curve, and decision curve analysis of machine learning model. RF, random forest; LightGBM, light gradient boosting machine; DT, decision tree; XGBoost, extreme gradient boosting; LR, logistic regression; KNN, *K*-nearest neighbors.

The predictive performance of the all-cause mortality risk model for CVD patients in both training and validation sets is summarized in Table 3. In the training set, the LightGBM algorithm demonstrated superior performance with an area under the ROC curve of 0.967, accuracy of 0.909, sensitivity of 0.906, specificity of 0.914, *F*1 score of 0.922, recall of 0.906, and area under the PR curve of 0.979. When applied to the validation set, LightGBM maintained robust performance with an area under the ROC curve of 0.793, accuracy of 0.720, sensitivity of 0.745, specificity of 0.684, *F*1 score of 0.760, recall of 0.745, and area under the PR curve of 0.852. Overall, LightGBM exhibited the best comprehensive performance among all tested algorithms.  Figure 6a,b illustrates the ROC analyses for both training and validation sets, respectively. As shown in Figure 6c, the LightGBM model achieved a net benefit of 0.5 when the threshold probability exceeded 60%, indicating good clinical utility. Furthermore, the calibration curve in Figure 6d demonstrates that the model's predicted probability closely aligns with the actual probability, confirming the high accuracy of the model. Moreover, when compared to benchmarks from published research [10], the LightGBM machine learning model achieved an AUC (95% confidence interval) of 0.967 (0.958–0.977), which is substantially higher than the Framingham risk score with an AUC (95% confidence interval) of 0.56 (0.43–0.69) and the SCORE system with an AUC (95% confidence interval) of 0.60 (0.46–0.73).

### 3.6. Interpretation of Machine Learning Models

**Figure 7:**
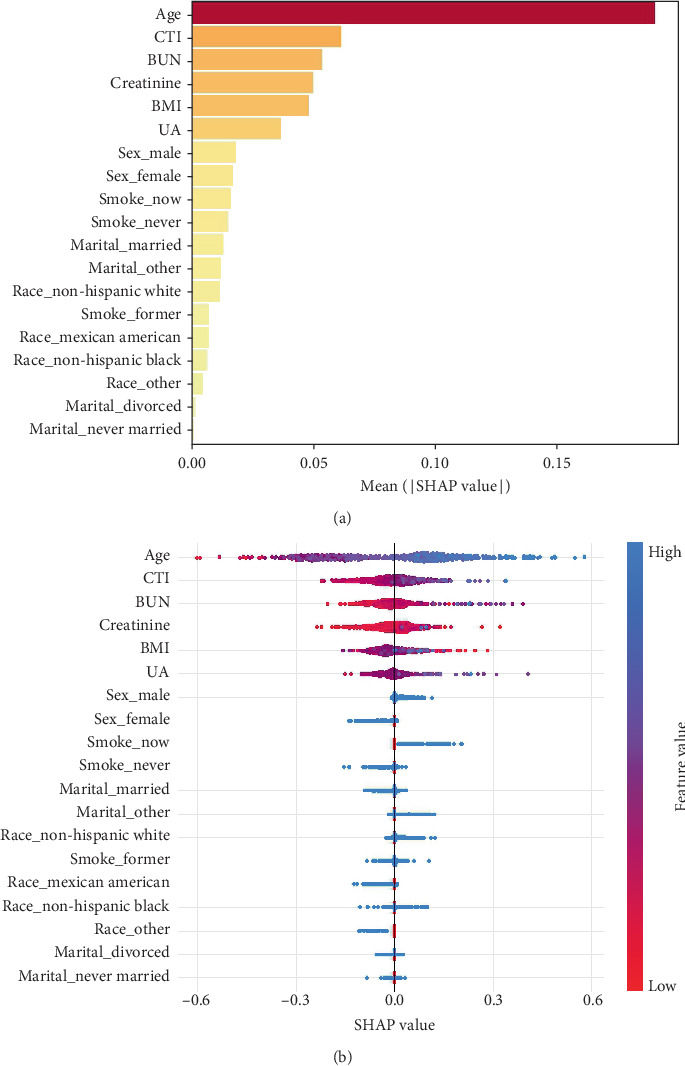
SHAP diagram of RF model. (a) SHAP value ranking of the variables in the model. (b) SHAP honeycomb diagram of the LightGBM model.

We conducted a SHAP analysis on the LightGBM model to assess the importance of each feature and its impact on model predictions, with results presented in Figure 7a,b. The analysis revealed that age is the most significant variable, showing the highest SHAP value and serving as a critical determinant for the risk of all-cause mortality in CVD patients. CTI ranks second among all variables, following age, and has a notable effect on the all-cause mortality risk in CVD patients. The importance of the other variables decreases progressively.

**Figure 8:**
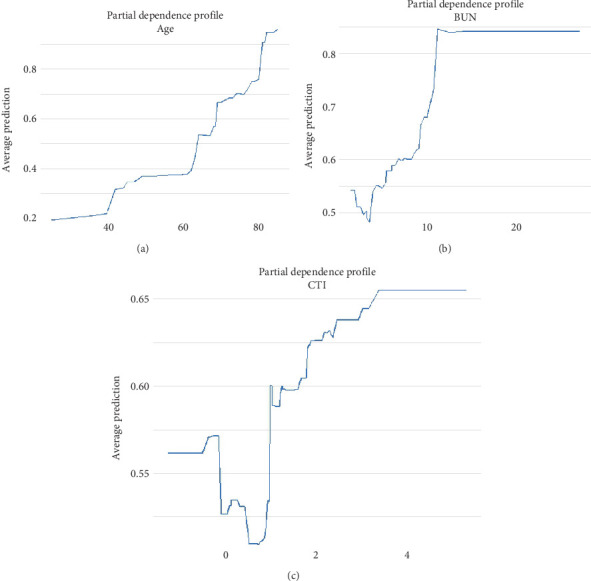
Partial dependence plots of LightGBM model for predicting all-cause mortality risk. (a) Age dependence profile, (b) BUN dependence profile, and (c) CTI dependence profile.

Furthermore, the partial dependence plots for the top three continuous variables displayed in Figure 7a are shown in Figure 8. According to Figure 8a, the curve indicating the relationship between age (*x*-axis) and all-cause mortality risk in CVD patients (*y*-axis) reveals a critical point at 60 years; beyond this threshold, the risk of mortality significantly increases. Similarly, Figure 8b,c illustrates that when the critical value for BUN is set at 10 and the critical value for CTI at 1, there is a notable rise in all-cause mortality risk. Therefore, targeted management of these critical values, as suggested by the partial dependence plots, has the potential to reduce the all-cause mortality risk in CVD patients, as age, BUN, and CTI all show a positive correlation with mortality risk.

## 4. Discussion

CVD is one of the leading causes of death worldwide, with its incidence and mortality rates continuing to rise, posing significant challenges to public health. According to statistics from the World Health Organization, CVD accounts for approximately 17 million deaths each year, representing 31% of global fatalities. The etiology of CVD is complex, involving a variety of factors including genetics, environment, and lifestyle. In recent years, inflammatory responses and metabolic abnormalities have been recognized as important pathological mechanisms of CVD [16, 17]. By identifying and managing risk factors associated with CVD, clinicians can effectively reduce the risk of cardiovascular events in patients. For example, controlling traditional risk factors such as hypertension, hyperlipidemia, and diabetes [18, 19], along with lifestyle improvements like increased physical activity and healthier diets, has been shown to significantly lower the incidence of CVD [20, 21]. Therefore, in-depth research into the pathogenesis of CVD and its related biomarkers is crucial for improving patient prognosis and reducing mortality rates.

CRP is an acute-phase reactant synthesized by the liver, with levels significantly elevated during inflammation, infection, and tissue damage. An increase in CRP is recognized as a marker of systemic inflammatory response; in recent years, it has gained widespread attention as a biomarker for CVD. Studies have demonstrated a close relationship between elevated CRP levels and increased risks of atherosclerosis, myocardial infarction, and other cardiovascular events [22]. CRP not only reflects systemic inflammatory status but may also exert direct effects on cardiovascular health by promoting endothelial dysfunction and the progression of atherosclerosis [23]. Furthermore, CRP levels are positively correlated with the incidence of cardiovascular events, particularly among high-risk populations [24, 25]. The TyG is a simple metric calculated as the product of fasting TGs and fasting glucose levels, and it has recently been recognized as an effective tool for assessing insulin resistance [26]. The method for calculating the TyG index is straightforward and easily accessible, allowing for broad application in clinical practice. Research indicates that the TyG index is closely associated with the risk of CVD, especially in patients with metabolic syndrome [27]. An increased TyG index is significantly correlated with the occurrence of cardiovascular events and demonstrates superior predictive capacity compared to traditional insulin resistance markers [28]. Moreover, the TyG index is not only related to cardiovascular events but is also closely linked to other metabolic abnormalities such as obesity and diabetes, making it an important indicator for assessing cardiovascular risk.

The assessment of prognosis in patients with CVD is a critical aspect of clinical management. Traditional prognostic evaluation methods primarily rely on clinical characteristics and routine biochemical markers; however, these approaches are somewhat limited in their ability to provide individualized risk assessments. In recent years, integrating inflammatory markers (such as CRP) with metabolic indicators (like the TG–glucose index), TyG for a comprehensive evaluation has been shown to enhance the accuracy of prognostic predictions [29]. Furthermore, with the advancement of big data and machine learning technologies, predictive models based on various clinical and biochemical parameters have become a research focus. These models can identify potential risk factors and provide support for clinical decision-making, ultimately improving patient management and outcomes [30]. By integrating CRP, TyG, and other relevant variables, clinicians can more accurately assess patient risk and formulate personalized treatment plans.

In this study, we included 1429 participants with an average age of 64.45 years, of whom 55.67% were male. The average age in the mortality group was significantly higher than that in the survival group, indicating that age is an important prognostic factor for patients with CVD. Moreover, our research found that levels of creatinine and BUN were significantly elevated in the mortality group (*p* < 0.0001), aligning with other studies that have highlighted the relationship between renal function and cardiovascular risk, suggesting that decline in renal function may be closely associated with the prognosis of CVD patients [31]. Notably, this study places a unique emphasis on the levels of cardiac troponin I, revealing that CTI levels were significantly higher in the mortality group compared to the survival group. This finding provides new evidence for the potential role of CTI as a prognostic indicator.

In this study, we employed a weighted multivariable Cox proportional hazards regression model to assess the independent association between CTI and mortality risk. The results demonstrated a positive correlation between CTI levels and all-cause mortality risk in CVD patients (HR = 1.24, 95% CI: 1.07–1.44, *p* = 0.004), which aligns with previous research that recognized CRP and TyG as cardiovascular risk markers. Notably, the highest quartile (Q4) of CTI significantly increased the all-cause mortality risk in CVD patients (HR = 1.38, 95% CI: 1.04–1.84, *p* = 0.03), indicating the importance of CTI in the prognostic evaluation of CVDs. Our study deliberately included uric acid levels in the adjustment model based on its important role in the pathogenesis of CVD and its close relationship with inflammation and oxidative stress. Although our adjustment model did not include all variables showing statistical differences between the survival and death groups at baseline (such as creatinine, BUN, alcohol use, and smoking status), our covariate selection strategy aimed to balance comprehensiveness and model parsimony, avoiding overfitting and multicollinearity among variables. In preliminary analyses, we attempted to include these additional variables for sensitivity analysis, and the results showed that the main association patterns remained unchanged, which enhances the reliability of our current model results. This study further explored the nonlinear relationship between CTI and both all-cause mortality and cardiovascular mortality, providing a more nuanced risk assessment perspective that is relatively uncommon in the existing literature. In subgroup analyses, we stratified participants based on factors such as age, sex, race, PIR, hypertension, and diabetes and found that the relationship between CTI and all-cause mortality risk remained consistent across all subgroups, with no significant interactions observed (*p* > 0.05). These findings suggest the broad applicability of CTI as a prognostic indicator. By analyzing various subgroups, this study confirmed the consistency of CTI across different populations, supporting its potential for clinical application. This discovery underscores the reliability of CTI in diverse contexts, potentially establishing it as a vital prognostic tool in clinical practice. Interestingly, while our adjusted Cox regression models demonstrated significant associations between CTI and all-cause mortality, the unadjusted Kaplan–Meier analysis showed only a nonsignificant trend (*p* = 0.515). This highlights the importance of accounting for confounding variables in our study population, as the relationship between CTI and mortality becomes more apparent after adjusting for relevant clinical and demographic factors. This phenomenon is not uncommon in complex epidemiological studies where risk factors may interact and confound each other.

In the comparison of machine learning models, this study showcased the performance of various algorithms in predicting all-cause mortality among patients with CVD, particularly focusing on the application of KNN and LightGBM algorithms. Compared to previous studies, this research provided a detailed evaluation of model effectiveness using specific performance metrics such as the area under the ROC curve and accuracy, revealing that LightGBM outperformed KNN in the validation set. However, the study also noted the potential for overfitting in the KNN algorithm within the training set, indicating that careful consideration of overfitting risks is essential when selecting a model. This finding underscores the importance of the differences in performance between validation and training sets during the development of machine learning models, reminding researchers to take into account the generalizability of the models when making selections.

Through SHAP analysis, this study evaluated the importance of each feature variable in the LightGBM model and its contribution to model predictions. The results indicated that age was the most important variable, possessing the highest SHAP value, and was identified as a significant risk factor for all-cause mortality in patients with CVD. This finding is consistent with existing research, highlighting the central role of age in the prognosis of CVDs [32, 33]. The CTI ranked third among all variables in terms of importance, following age and BUN, further confirming its independent role in assessing all-cause mortality risk in CVD patients. Additionally, the study utilized partial dependence plots to analyze the threshold values for age, BUN, and CTI, revealing that all-cause mortality risk in CVD patients significantly increased when age exceeded 60 years, BUN surpassed 10 mg/dL, and CTI exceeded 1. This finding provides important clinical reference points, suggesting that healthcare providers should focus on these key indicators when managing CVD patients.

The machine learning models developed in this study not only demonstrate excellent technical metrics but, more importantly, address key gaps in actual clinical practice. First, in resource-limited healthcare environments, precise risk stratification can help clinicians prioritize patients who most need close monitoring and aggressive intervention. Second, compared to traditional risk scores, our model more accurately reflects patients' inflammatory status and potential cardiovascular risk by integrating CTI as a novel biomarker, especially in borderline populations where traditional risk factor assessment yields unclear results. We propose these models be used as clinical decision support tools rather than replacements for clinical judgment. In practical application, physicians can input relevant metrics through a simple web interface or mobile application to obtain patients' risk predictions and risk category classifications. This information can be used in physician–patient communication to help patients understand their risk and improve treatment adherence.

Despite the important findings presented in this study, there are several limitations to consider. Firstly, this research is based on NHANES data and is thus a retrospective observational study. This design may introduce selection bias and information bias, limiting the clarity of causal relationships. Therefore, future prospective studies may be more effective in validating the relationship between CTI and all-cause mortality risk in CVD patients. Secondly, although the study included 1429 CVD patients, the selection of the sample may impact the generalizability of the results. If the sample is predominantly drawn from specific regions or populations, it may not accurately represent the broader CVD patient population. Thirdly, while potential confounders were adjusted for in the analysis, there may still be unidentified or inadequately controlled confounders that could influence the relationship between CTI and all-cause mortality risk. For instance, lifestyle factors (such as diet, exercise, and smoking) and the effects of other biomarkers may not have been fully accounted for. Fourthly, the study employed multiple machine learning models for prediction. Although the LightGBM model outperformed others, the choice of machine learning models and parameter tuning could affect the stability and reproducibility of the results. Additionally, the risk of overfitting is highlighted by differences in performance between the training and validation sets, indicating a need for further validation and optimization. Lastly, while the SHAP analysis provides valuable insights into model interpretability, its results depend on the accuracy of the model and the appropriateness of feature selection. The interpretation of important features may be influenced by other potential factors, thus necessitating cautious interpretation of the SHAP values' results.

In conclusion, this study has developed and validated a machine learning model that integrates CTI and traditional cardiovascular risk factors, not only demonstrating superior predictive accuracy but also providing a practical tool for precision medicine, helping clinicians perform targeted risk stratification, optimize follow-up strategies, and guide early interventions, potentially improving cardiovascular outcomes in high-risk populations. This model addresses limitations in existing risk assessment tools, particularly in evaluating the role of inflammation in CVD development, offering new perspectives for future individualized prevention and treatment strategies.

## Figures and Tables

**Table 1:** Baseline characteristics of participants.

**Variables**	**Total**	**Survival**	**Death**	**p** ** value**
Age, mean (SE)	64.45 (0.46)	57.13 (0.52)	70.50 (0.51)	< 0.0001
Creatinine, mean (SE)	97.13 (2.46)	83.45 (1.79)	108.42 (4.11)	< 0.0001
Uric acid, mean (SE)	359.93 (3.39)	349.34 (4.52)	368.68 (4.47)	0.002
BUN, mean (SE)	6.18 (0.10)	5.12 (0.10)	7.05 (0.15)	< 0.0001
TG, mean (SE)	1.87 (0.05)	1.92 (0.09)	1.83 (0.05)	0.39
TC, mean (SE)	4.97 (0.04)	4.98 (0.07)	4.96 (0.05)	0.77
HDL, mean (SE)	1.30 (0.01)	1.26 (0.02)	1.32 (0.02)	0.02
LDL, mean (SE)	2.86 (0.03)	2.90 (0.05)	2.83 (0.04)	0.31
BMI, mean (SE)	29.69 (0.21)	30.48 (0.33)	29.05 (0.27)	0.001
CRP, mean (SE)	0.57 (0.03)	0.44 (0.03)	0.67 (0.05)	< 0.0001
FPG, mean (SE)	6.46 (0.06)	6.24 (0.07)	6.64 (0.10)	< 0.001
HbA1c, mean (SE)	5.95 (0.03)	5.84 (0.04)	6.05 (0.04)	< 0.001
CTI, mean (SE)	1.06 (0.03)	0.98 (0.04)	1.12 (0.04)	0.01
Sex, % (SE)				0.8
Male	55.67 (0.03)	55.28 (2.31)	55.99 (1.77)	
Female	44.33 (0.03)	44.72 (2.31)	44.01 (1.77)	
Race, % (SE)				0.004
Mexican American	3.84 (0.01)	5.29 (1.12)	2.65 (0.59)	
Non-Hispanic Black	10.67 (0.01)	12.15 (1.38)	9.45 (1.09)	
Non-Hispanic White	78.03 (0.05)	72.76 (2.22)	82.39 (1.87)	
Other	7.45 (0.01)	9.79 (1.76)	5.52 (1.35)	
Marital, % (SE)				< 0.0001
Married	59.78 (0.04)	66.92 (2.89)	53.88 (2.19)	
Never married	4.29 (0.01)	5.10 (1.05)	3.62 (0.60)	
Divorced	10.31 (0.01)	10.38 (1.33)	10.25 (1.20)	
Unmarried but have/had partner	25.62 (0.02)	17.59 (2.39)	32.25 (1.92)	
Education, % (SE)				0.002
Less than high school	30.76 (0.02)	24.95 (2.35)	35.55 (2.3)	
High school or equivalent	26.60 (0.03)	25.29 (2.51)	27.68 (2.41)	
College or above	42.64 (0.03)	49.75 (3.01)	36.77 (2.36)	
Smoke, % (SE)				0.05
Never	37.13 (0.03)	38.42 (2.51)	36.08 (2.22)	
Former	41.48 (0.03)	37.45 (2.05)	44.80 (2.14)	
Now	21.39 (0.02)	24.14 (2.16)	19.12 (1.48)	
Alcohol, % (SE)				< 0.0001
Never	12.52 (0.01)	8.71 (1.74)	15.66 (1.58)	
Former	34.01 (0.03)	28.66 (2.45)	38.44 (2.29)	
Mild	36.27 (0.02)	40.52 (2.41)	32.75 (1.96)	
Moderate	7.50 (0.01)	8.87 (1.28)	6.36 (0.98)	
Heavy	9.70 (0.01)	13.24 (1.57)	6.78 (0.98)	
Hypertension, % (SE)				0.002
Yes	72.76 (0.05)	67.93 (2.61)	76.74 (1.92)	
No	27.24 (0.02)	32.07 (2.61)	23.26 (1.92)	
Diabetes, % (SE)				< 0.0001
Yes	34.30 (0.03)	27.82 (2.23)	39.65 (2.22)	
No	48.11 (0.03)	55.14 (2.59)	42.29 (2.30)	
Borderline	17.59 (0.02)	17.04 (1.59)	18.05 (1.62)	
PIR, % (SE)				< 0.0001
< 1.3	27.12 (0.02)	22.42 (1.98)	30.99 (2.21)	
1.3–3.5	40.85 (0.03)	33.41 (2.41)	46.99 (2.23)	
> 3.5	32.04 (0.02)	44.17 (2.82)	22.02 (1.98)	

*Note:* Dates are presented as mean (SE) or percentage (SE).

Abbreviations: BMI, body mass index; BUN, blood urea nitrogen; CRP, C-reactive protein; CTI, C-reactive protein–triglyceride–glucose index; FPG, fasting plasma glucose; HbA1c, glycosylated hemoglobin; HDLC, high-density lipoprotein; LDL, low-density lipoprotein; PIR, poverty income ratio; TC, total cholesterol; TG, triglyceride.

**Table 2:** Analysis of association between CTI and all-cause mortality.

**Variables**	**Model 1**		**Model 2**		**Model 3**	
	**HR (95% CI)**	**p**	**HR (95% CI)**	**p**	**HR (95% CI)**	**p**
All-cause mortality						
CTI	1.09 (0.99, 1.20)	0.08	1.30 (1.18, 1.44)	< 0.0001	1.24 (1.07, 1.44)	0.004
CTI category						
Q1	Ref	Ref	Ref	Ref	Ref	Ref
Q2	0.93 (0.73, 1.18)	0.54	0.92 (0.72, 1.19)	0.53	0.93 (0.74, 1.18)	0.55
Q3	1.17 (0.92, 1.48)	0.20	1.16 (0.91, 1.48)	0.24	1.06 (0.81, 1.39)	0.68
Q4	1.12 (0.89, 1.41)	0.32	1.59 (1.26, 2.01)	< 0.0001	1.38 (1.04, 1.84)	0.03
*p* for trend	0.13		< 0.0001		0.035	

*Note:* Model 1: no adjustments made. Model 2: adjusted for age, sex, and race. Model 3: adjusted for age, sex, race, smoking, alcohol, marital status, education, hypertension, diabetes, PIR, BMI, creatinine, UA, BUN, HbA1c, TC, HDL, and LDL.

Abbreviations: CI, confidence interval; HR, hazard ratio; Ref, reference.

**Table 3:** Performance of machine learning model in training set and validation set.

**Models**	**Accuracy**	**F**1	**Sensitivity**	**Specificity**	**Recall**	**ROC-AUC**	**PR-AUC**
Training set							
DT	0.741	0.768	0.722	0.768	0.722	0.826	0.884
RF	0.793	0.809	0.737	0.874	0.737	0.895	0.925
XGBoost	0.755	0.774	0.705	0.828	0.705	0.845	0.886
LightGBM	0.909	0.922	0.906	0.914	0.906	0.967	0.979
KNN	0.903	0.920	0.934	0.857	0.934	0.963	0.974
LR	0.765	0.795	0.766	0.764	0.766	0.840	0.888
Validation set							
DT	0.671	0.707	0.667	0.678	0.667	0.764	0.836
RF	0.702	0.718	0.639	0.793	0.639	0.801	0.841
XGBoost	0.697	0.715	0.639	0.782	0.639	0.799	0.838
LightGBM	0.720	0.760	0.745	0.684	0.745	0.793	0.852
KNN	0.664	0.721	0.729	0.569	0.729	0.734	0.803
LR	0.746	0.779	0.753	0.736	0.753	0.827	0.887

Abbreviations: DT, decision tree; KNN, *K*-nearest neighbors; LightGBM, light gradient boosting machine; LR, logistic regression; RF, random forest; XGBoost, extreme gradient boosting.

## Data Availability

The data that support the findings of this study are available from the corresponding author upon reasonable request.

## Nomenclature

CTI: C-reactive protein–triglyceride–glucose index
CVD: cardiovascular disease
ML: machine learning
CRP: C-reactive protein
TyG: triglyceride–glucose index
BMI: body mass index
BUN: blood urea nitrogen
TG: triglyceride
TC: total cholesterol
HDL: high-density lipoprotein
LDL: low-density lipoprotein
PIR: poverty income ratio
HbA1c: glycosylated hemoglobin
FPG: fasting plasma glucose
NHANES: National Health and Nutrition Examination Survey
RF: random forest
LightGBM: light gradient boosting machine
DT: decision tree
XGBoost: extreme gradient boosting
LR: logistic regression
KNN: *K*-nearest neighbors
SHAP: Shapley's Additive Explanations
